# Quantitative and Qualitative Control of Wheat Flour Dough's Mechanical Properties via Dietary Protein Addition

**DOI:** 10.1002/fsn3.72564

**Published:** 2026-11-04

**Authors:** Jingxian An, Owen Jones, Senay Simsek, Kristin Whitney, Bruce Hamaker, Yuan Yao

**Affiliations:** ^1^ Whistler Center for Carbohydrate Research, Department of Food Science Purdue University West Lafayette Indiana USA

**Keywords:** dietary protein, mechanical properties, permutation importance, Plant‐Based Protein, regression and classification models, wheat flour dough

## Abstract

Dietary protein fortification enhances bakery nutrition, but its impact on dough mechanics remains unclear. To address this, 432 formulations were designed using eight proteins (up to 70.6% substitution), seven lipid configurations, and salt, with water adjusted (38%–159% baker's percentage). Texture profile analysis (10 mm compression distance) and dynamic rheometry (0.1–80 Hz at 0.5% strain) yielded 19 continuous and tertile‐discretized mechanical target variables. Parallel regression and classification workflows were developed to predict continuous values and tertile‐defined categories (low, medium, and high). Models included Ridge regression for continuous prediction, logistic regression for classification, and Random Forest and Gradient Boosting for both tasks, with performance evaluated using five‐fold cross‐validation. Both paradigms generally predicted rheological properties with higher accuracy (*R* ^2^ up to 0.87, classification accuracy up to 89.3%) than textural attributes (*R* ^2^ up to 0.78, accuracy up to 82.9%). Permutation importance revealed that soy protein, a pea‐quinoa blend, and bread flour were leading predictors, whereas lipids and salt showed low average predictive weight. Rheological regression models required eight to 10 predictors to retain ≥ 90% of full‐model *R* ^2^, whereas hardness, gumminess, chewiness, and resilience required four. Casein's normalized predictive importance was 42.1% higher in classification than in regression, indicating task‐dependent relevance. Rather than demonstrating biophysical causation, this research establishes an internally validated predictive framework. These findings advance formulation science by offering a mathematical basis to quantitatively screen and rationally design composite dough formulations. This predictive tool may support high‐plant‐protein food development by prioritizing formulations for experimental testing within the investigated domain.

## Graphical abstract

Regression and classification models linked 432 wheat flour–protein dough formulations to 19 mechanical properties. Rheological properties were generally more predictable than texture properties, with cross‐validated *R* ^2^ reaching 0.865 and classification accuracy reaching 89.3%. Soy protein, the pea–quinoa protein blend, and bread flour were leading predictors across both tasks. Selected texture properties retained ≥ 90% of full‐model regression performance with four predictors, whereas rheological properties required eight to 10. These predictive associations support protein selection and dosage as priorities for formulation screening within the tested formulation space.

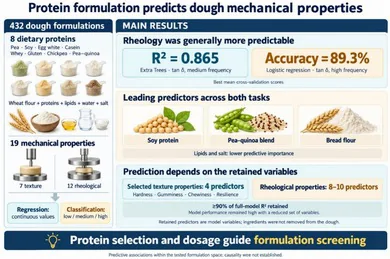

## 1 Introduction

With growing public attention to healthy eating, health‐conscious consumers have increasingly sought high‐protein food products to boost their health and wellness (Cavalheiro et al. 2020). For instance, dairy protein may indirectly improve metabolic health by aiding weight loss and reducing fat mass through enhanced satiety (McGregor and Poppitt 2013). Similarly, protein intake—both quantity and quality—plays an important role in growth, neurodevelopment, and long‐term health during the first 2 years of life (Michaelsen and Greer 2014). Given the importance of dietary protein, an increasing number of food products are now being fortified with it, with bakery products receiving particular attention. For example, soy protein concentrate was blended with wheat flour at varying mass ratios to develop high‐protein precooked noodle samples (Yoon et al. 2022). In a related approach, Matos et al. (2014) incorporated soy protein isolate, pea protein isolate, egg white protein, and casein into rice flour to produce gluten‐free muffins, and found that the type of protein added differentially affected the rheological properties of the batter and the texture of the muffins.

Besides health outcomes, dietary protein addition also brings many other benefits. For example, the addition of soy protein concentrate to precooked noodles can improve the dough stability of wheat flour (Yoon et al. 2022). Meanwhile, pea protein addition can increase the dough's extensibility and tenacity (García‐Segovia et al. 2020). Moreover, Song et al. (2018) found that breads enriched with 7% whey protein hydrolysate, or with 4% whey protein hydrolysate combined with 4% soy protein isolate, were preferred by consumers. However, it should be noted that the addition of dietary protein to bakery products can also alter processability. This occurs because changes in protein content and composition directly affect the dough's mechanical properties, which determine how the dough behaves during mixing, shaping, and baking. Similarly, Ziobro et al. (2013) found that the use of albumin, collagen, and pea, lupine, and soy protein in wheat flour dough can significantly influence their rheological properties (a category of mechanical properties). Similarly, Pořízka et al. (2023) found that 5% whey protein isolate substitution in wheat flour baked goods resulted in superior mechanical properties compared to formulations without protein addition. Moreover, Tang and Liu (2017) found that the addition of soy protein increased the dough's loss modulus and tan *δ*.

Crucially, these rheological shifts are fundamentally governed by native wheat storage proteins (polymeric glutenins and monomeric gliadins), which establish the viscoelastic gluten network (Zhou et al. 2026). Within this matrix, high‐molecular‐weight glutenin subunits (HMW‐GS) utilize disulfide cross‐links and “loop‐train” conformational motifs to form the primary elastic units (Zhou et al. 2026). When exogenous non‐wheat proteins are incorporated, they dilute these native storage proteins and aggressively compete for water (Dotto et al. 2025). This steric hindrance and hydration competition disrupt the disulfide‐bonded network (Dotto et al. 2025; Zhou et al. 2026). Consequently, this molecular‐level disruption leads to a severe deterioration of gluten network integrity, manifesting instrumentally as increased dough brittleness, reduced chewiness, and a loss of textural elasticity (Dotto et al. 2025).

Despite these valuable individual insights, several limitations remain in understanding and predicting the mechanical properties of protein‐fortified composite doughs. First, studies such as those by Yoon et al. (2022) and Matos et al. (2014) demonstrate that protein addition can alter dough stability, batter rheology, and product texture, but their findings concern specific protein types, substitution levels, and product matrices. They therefore provide limited guidance for predicting mechanical responses across broader formulation conditions. Second, differences in the mechanical parameters assessed and hydration protocols used can complicate comparisons among studies, particularly because proteins differ in their water absorption capacities. Third, conventional trial‐and‐error formulation approaches require substantial experimental effort when multiple ingredients are varied and may not systematically characterize nonlinear formulation–property relationships. Consequently, there remains a need for an integrated predictive framework that combines continuous quantitative estimation through regression with categorical screening through classification across multiple dough mechanical properties.

To address these gaps, this study establishes a systematic predictive and screening framework using 432 dough formulations. The experimental design incorporates eight plant and animal protein ingredients, with substitution levels up to 70.6%, seven lipid configurations, and salt, while empirically adjusting water to obtain workable doughs. Parallel regression and classification workflows were developed to predict 19 mechanical properties spanning dynamic rheology and texture, using continuous measurements and tertile‐defined categories, respectively. Furthermore, permutation‐based feature importance analysis and sequential predictor removal were implemented to evaluate the predictive relevance of individual ingredients and assess model performance as the number of predictors decreased. By combining multiple mechanical outcomes with complementary prediction tasks, this work extends formulation‐specific observations toward quantitative screening within the investigated formulation space and provides guidance for prioritizing experimental testing of protein‐fortified composite doughs.

## 2 Material and Methods

### 2.1 Dough Formulation and Measurement Overview

To construct a robust and structurally unbiased predictor matrix for our machine learning models, the 432 distinct dough samples were engineered using a highly structured, orthogonal combinatorial framework (Table 1). The complete dataset is systematically partitioned into three distinct functional cohorts:

- 12 Protein‐Free Controls: Formulated solely with wheat flour and diverse lipid configurations to establish the baseline mechanical profiles of the starch‐gluten matrix without protein supplementation
- 224 Mono‐Protein Formulations: Structured around an 8 × 7 × 4 full factorial design. For each of the eight individual dietary protein sources, we applied seven systematic lipid combinations (three pure oils/fats and four blended configurations). Under each lipid scenario, four distinct flour‐to‐lipid‐to‐water ratios were developed, yielding precisely 28 unique observations for each protein type (8 × 28 = 224 total).
- 196 Dual‐Protein Blends: Arranged via a 28 × 7 factorial grid. This cohort evaluated all possible pairwise protein mixtures (C (8, 2) = 28 binary combinations) crossed with the same seven lipid configurations (28 × 7 = 196 total).

**TABLE 1:** Combinational layout and factorial structure of the 432 sample observations.

Dataset partition	Protein system	Fat/lipid configuration	Ratios per condition	Sample count	Frequency per protein
Control (protein‐free)	None (wheat flour)	12 varied configurations	—	12	N/A
Mono‐Protein	8 single sources	7 configurations (3 pure +4 blends)	4 formulation levels	224	*n* = 28 per protein
Dual‐Protein	28 pairwise blends	7 configurations (3 pure +4 blends)	1 fixed level	196	*n* = 49 per protein
Full dataset	8 dietary proteins	7 systematic fat configurations (3 pure +4 blends)	—	432	Strictly *n* = 77 for each protein

A major advantage of this combinatorial architecture is the uniform distribution of proteins across the dataset: each of the eight dietary proteins is represented in exactly *n* = 77 formulations (28 single‐protein + 49 binary‐protein), which eliminates frequency‐related bias in the discrete predictor space. Although the categorical factors were strictly balanced, the continuous parameters (protein substitution ceilings, lipid ratios, and salt percentages) were set according to the physical constraints described in our previous work (An et al. 2026). The maximum incorporation levels for individual proteins were 52.3% for pea, 61.1% for soy, 39.7% for egg white, 51.7% for casein, 20.0% for whey, 60.0% for gluten, 28.6% for chickpea, and 49.7% for pea‐quinoa, and combined binary‐protein substitution reached up to 70.6%. Because of the markedly different hydration capacities of these proteins, water addition was adjusted for each formulation to ensure proper handling properties, preventing both fragmentation and surface stickiness during rheological and textural testing. Consequently, the water‐to‐flour ratio ranged from 0.55–0.71 in protein‐free controls to 1.08–1.59 in formulations containing casein or concentrated pea/soy blends. The complete baker's percentages for all 432 formulations are provided in Supplementary Table 1.

To systematically profile the mechanical behavior across all formulations, standardized instrumental protocols for dynamic oscillatory rheology and texture profile analysis (TPA) were implemented directly. Dynamic rheological properties of the 30‐g hand‐rounded dough samples were evaluated at 25°C using a stress‐controlled rotational rheometer (Discovery HR‐2, TA Instruments, New Castle, DE, USA) fitted with a 40 mm stainless‐steel parallel‐plate geometry (measurement gap: 1000 μm; loading gap: 45,000 μm; trim gap offset: 50 μm) under Peltier temperature control. Small‐amplitude oscillatory frequency sweeps were executed across a frequency range of 0.1–80 Hz at a controlled strain of 0.5% (within the linear viscoelastic region), collecting 22 angular frequency points. These data were partitioned into low‐, medium‐, and high‐frequency regions according to deformation timescale behavior, yielding 12 rheological variables: storage modulus (*G*′), loss modulus (*G*′′), loss tangent (tan *δ*), and complex viscosity (η*) across each of the three frequency regions. Textural profiles (hardness, adhesiveness, springiness, cohesiveness, gumminess, chewiness, and resilience; 7 TPA parameters) were concurrently measured on a texture analyzer (TA.XT Plus, Stable Micro Systems, Godalming, UK) at 25°C, using pre‐test, test, and post‐test speeds of 3.00, 1.00, and 1.00 mm/s, respectively, with the probe set to compress the dough by 10.000 mm, a trigger force of 5.0 g, and auto tare mode.

### 2.2 Machine Learning Methods

#### 2.2.1 Regression Model and Classification Model

To comprehensively predict the mechanical properties of wheat flour–protein dough, both regression and classification models were developed and evaluated in parallel, allowing the mechanical properties to be predicted as continuous values (regression) as well as categorical levels (classification).

For regression analysis, five candidate models were employed to predict each mechanical property as a continuous variable. Ridge regression applies L2 regularization to linear relationships, offering a simple and interpretable baseline (Gillariose et al. 2026). Support vector regression with a radial basis function kernel (SVR_RBF) captures nonlinear relationships by mapping inputs into a higher‐dimensional space (Aldhafferi 2024). Random forest and extra trees regression are ensemble methods that aggregate predictions from multiple decision trees, with extra trees introducing additional randomness in split selection to reduce overfitting (Galelli and Castelletti 2013). Histogram‐based gradient boosting regression builds trees sequentially, with each tree correcting the errors of its predecessors, making it well‐suited for capturing complex, nonlinear feature interactions (Sichani et al. 2025). Together, these models span linear, kernel‐based, and tree‐based ensemble paradigms, enabling a comprehensive comparison of predictive performance under different algorithmic assumptions.

For classification analysis, each continuous mechanical property was discretized into three categorical levels. Formulations were ranked according to their measured values and classified into three approximately equal groups: the lowest one‐third as “low,” the middle one‐third as “medium,” and the highest one‐third as “high.” To prevent data leakage, these tertile boundaries were calculated independently within each training fold and subsequently applied to classify the corresponding validation fold. In this study, five classification models were evaluated, mirroring the regression model structure: logistic regression, a linear probabilistic classifier (Janani et al. 2017); support vector classification with a radial basis function kernel (SVC_RBF), which separates classes using nonlinear decision boundaries (Razaque et al. 2021); random forest and extra trees classification, ensemble tree‐based classifiers differing in their degree of split randomness; and histogram‐based gradient boosting classification, which sequentially builds trees to iteratively improve classification accuracy.

For both regression and classification tasks, all models were implemented within standardized preprocessing pipelines, incorporating median imputation for missing values and feature standardization (z‐score normalization) prior to model fitting. For regression models, the target variable was additionally standardized during training and transformed back to its original scale for prediction, ensuring stable model performance across mechanical properties with different measurement units and value ranges. Each mechanical property was modeled independently using 5‐fold cross‐validation, in which the dataset was randomly partitioned into five subsets; in each iteration, four subsets (80% of the data) were used for model training and the remaining subset (20%) for validation, with this process repeated five times so that every subset served as the validation set exactly once. For classification tasks, folds were stratified to preserve the proportion of low, medium, and high categories across training and validation subsets. All candidate models were evaluated using identical cross‐validation fold assignments to ensure a fair and consistent comparison of predictive performance across regression and classification tasks.

#### 2.2.2 Feature Selection Methods

To identify the relative contribution of each food ingredient to dough mechanical properties, a permutation importance‐based feature selection procedure was performed. For each of the 19 mechanical properties, ingredient importance was assessed independently using permutation importance under repeated 5‐fold cross‐validation. Within each validation fold, an Extra Trees regressor and an Extra Trees classifier were trained on the remaining ingredients, and the importance of each ingredient was calculated by measuring the decrease in model performance (*R* ^2^ for regression; balanced accuracy for classification) after randomly permuting that ingredient's values while holding all other ingredients constant. Extra Trees was selected as the sole model for permutation importance calculation because it can capture nonlinear relationships and interactions among ingredients without requiring pre‐specified functional forms, and using a single consistent model throughout the sequential removal process ensures that importance rankings remain internally comparable across removal steps, independent of which model performed best for a given mechanical property. Importance scores were normalized within each property to sum to one, ensuring comparability across properties with different measurement scales. For each ingredient, importance values were averaged across all 19 mechanical properties to obtain an overall ranking. Regression‐based and classification‐based importance were reported separately throughout, allowing the relative contribution of each ingredient to be examined independently for continuous and categorical prediction tasks.

#### 2.2.3 Sequential Ingredient Removal

Building on this importance ranking, a sequential ingredient‐removal procedure was subsequently conducted to evaluate how model performance changed as less important ingredients were progressively excluded. At each removal step, permutation importance was recalculated using only the remaining ingredients, and the ingredient with the lowest importance was removed; this process was repeated iteratively until only one ingredient remained. Because ingredient importance may shift after the removal of correlated or interacting ingredients, importance was recalculated at every step rather than being fixed in advance. Regression and classification removal orders were derived independently, as the ranking of ingredient importance could differ between continuous and categorical prediction tasks. At each removal step, the best‐performing regression model and the best‐performing classification model identified in Section 2.2.1 were retrained and evaluated using the same cross‐validation folds, allowing the change in predictive performance to be tracked as a function of the number of ingredients retained.

## 3 Results and Discussion

### 3.1 Quantitative Control of Mechanical Properties

#### 3.1.1 Regression Models Predicting Wheat Flour Dough's Mechanical Properties

Regression models utilizing Ridge, SVR_RBF, random forest, extra trees, and histogram‐based gradient boosting were used to predict wheat flour–protein dough's mechanical properties, with predictive performance evaluated across all 19 texture and rheological properties using mean cross‐validated *R* ^2^ (Table 2). According to Table 2, Ridge regression achieved the highest average predictive performance across the 19 properties, performing particularly well for rheological properties. It achieved the highest *R* ^2^ for storage modulus and complex viscosity across all frequency regions and loss modulus at low and medium frequencies. However, model performance was property‐dependent: Extra Trees achieved the highest *R* ^2^ for tan(δ) across all frequency regions, reaching 0.865 at medium frequency, whereas SVR_RBF performed best for loss modulus at high frequency. Across all five models, predictive performance for storage modulus, loss modulus, and complex viscosity generally decreased from low to high frequency, while the opposite trend was observed for tan(δ), indicating that frequency‐related differences in predictability depended on the rheological response. In contrast, predictive performance was considerably lower for several textural properties, with adhesiveness, springiness, and cohesiveness showing relatively low *R* ^2^ values across all models. Nonlinear models, including Extra Trees and SVR_RBF, achieved higher *R* ^2^ values for specific attributes such as cohesiveness (*R* ^2^ = 0.402) and gumminess (*R* ^2^ = 0.610), respectively. Resilience was an exception to the generally lower predictability of textural properties, with *R* ^2^ values ranging from 0.697 to 0.778. Adhesiveness was also difficult to predict (*R* ^2^ = 0.244–0.413), potentially because it reflects both the dough's viscoelastic behavior and its interaction with the contacting surface. Previous research has shown that dough adhesion depends on contact‐surface characteristics and contact time, suggesting that factors beyond formulation composition may contribute to its limited predictability (Laukemper et al. 2021).

**TABLE 2:** Predictive performance (mean cross‐validated *R* ^2^) of five regression models for wheat flour–protein dough's texture and rheological properties.

Mechanical properties	Ridge	SVR_RBF	Random forest	Extra trees	Hist gradient boosting
Hardness	0.640	**0.648**	0.603	0.634	0.562
Adhesiveness	**0.413**	0.277	0.336	0.337	0.244
Springiness	0.258	0.251	0.233	**0.278**	0.249
Cohesiveness	0.351	0.376	0.336	**0.402**	0.321
Gumminess	0.527	0.610	0.553	**0.591**	0.528
Chewiness	0.409	**0.519**	0.386	0.432	0.403
Resilience	0.697	0.777	0.753	**0.778**	0.751
Storage modulus_Low	**0.695**	0.642	0.581	0.635	0.560
Storage modulus_Medium	**0.651**	0.627	0.569	0.600	0.559
Storage modulus_High	**0.612**	0.591	0.492	0.512	0.493
Loss modulus_Low	**0.654**	0.582	0.518	0.524	0.490
Loss modulus_Medium	**0.601**	0.572	0.467	0.504	0.437
Loss modulus_High	0.492	**0.534**	0.412	0.472	0.284
Tan (δ)_Low	0.740	**0.763**	0.723	0.773	0.680
Tan (δ)_Medium	0.814	0.816	0.819	**0.865**	0.652
Tan (δ)_High	0.859	0.838	0.840	**0.861**	0.698
Complex viscosity_Low	**0.701**	0.653	0.581	0.613	0.556
Complex viscosity_Medium	**0.666**	0.631	0.558	0.603	0.539
Complex viscosity_High	**0.612**	0.596	0.465	0.518	0.467

*Note:* The highest values within each row are bolded.

These differences likely reflect, in part, the deformation conditions used to measure each property type. Rheological properties were measured under small‐strain oscillatory conditions, which probe the intrinsic viscoelastic behavior of the protein–starch network—a structure influenced by protein type and amount—potentially resulting in stronger, more predictable relationships with formulation composition. Textural properties, in contrast, were obtained through large‐deformation compression testing, which captures more complex mechanical responses that may be influenced by factors beyond formulation composition, such as structural heterogeneity and measurement variability. The comparatively higher performance of nonlinear models for several textural properties suggests that these responses may involve more complex formulation–property relationships. However, these explanations remain tentative because predictive performance alone does not establish the underlying mechanisms.

#### 3.1.2 Ingredient Importance Ranking for Predicting Mechanical Properties

Permutation importance analysis revealed marked differences in how individual food ingredients contributed to the model's predictive capacity regarding dough mechanical properties (Figure 1). Because importance values were normalized within each mechanical property and subsequently averaged across all 19 outputs, the ranking represents overall predictive relevance rather than an identical contribution to every individual property. Higher scores indicate greater predictive reliance on an ingredient, but do not indicate the direction of its effect. Based on regression metrics, soy protein was identified as the most dominant predictive feature, exhibiting an importance score substantially higher than any other formulation component. This prominent predictive association may partly reflect formulation changes discussed in previous studies, including gluten dilution, hydration competition, and soy–gluten protein interactions (Zhang et al. 2025). Similarly, Roccia et al. (2009) reported that soy protein isolate weakened hydrated gluten through structural interference and reduced water availability for gluten network formation. These experimental observations provide plausible explanations for the high predictive importance of soy protein, although the present analysis does not directly establish these mechanisms. Bread flour and the pea‐and‐quinoa protein mixture followed soy protein with nearly identical importance scores, indicating comparable overall predictive relevance rather than equivalent functional effects. Their similar scores indicate comparable overall predictive relevance rather than equivalent functional effects. Bread flour provides the wheat flour matrix, whereas the pea‐and‐quinoa mixture introduces additional protein ingredients whose incorporation may alter matrix composition and hydration behavior. Because this mixture was treated as a single formulation variable, its importance cannot be attributed separately to pea and quinoa or interpreted as evidence of synergism between them. Pea protein ranked fourth, followed by gluten protein and chickpea protein; collectively, these top six protein‐related ingredients accounted for the vast majority of the total regression importance. The lower rankings of gluten and chickpea proteins relative to soy and pea proteins further indicate that predictive importance varied among the tested protein ingredients, rather than being determined solely by their inclusion as protein sources.

**FIGURE 1:**
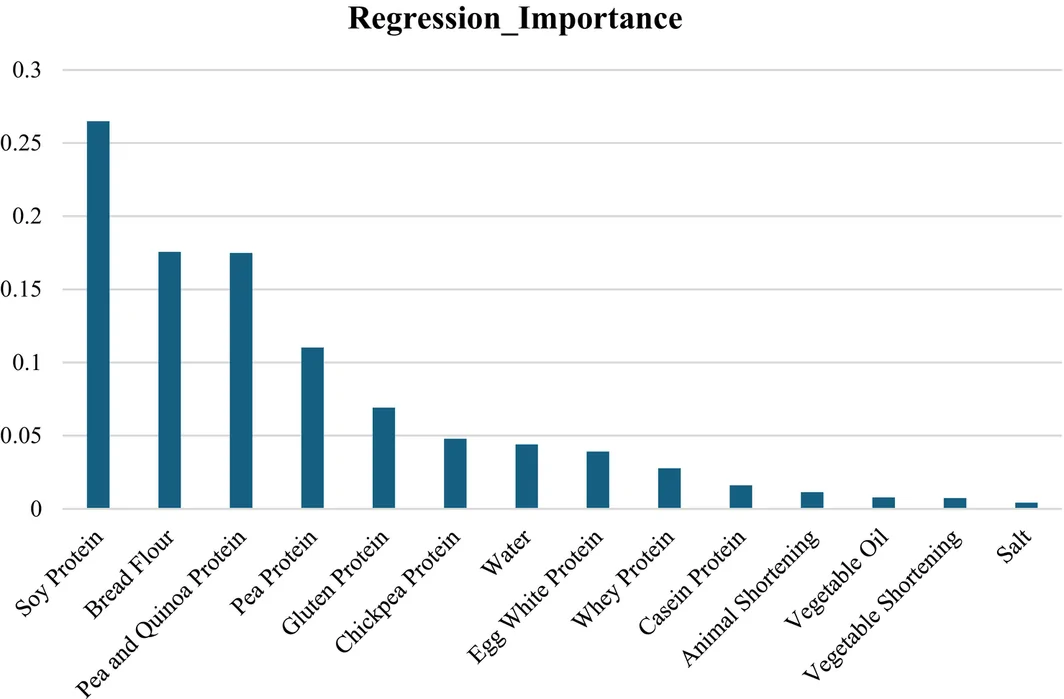
Regression‐based permutation importance of individual food ingredients in predicting wheat flour dough's mechanical properties. Importance values were normalized within each mechanical property and averaged across all 19 texture and rheological properties to obtain the overall ranking shown.

By comparison, water, egg white protein, whey protein, and casein protein showed relatively modest importance, while lipid‐related ingredients (animal shortening, vegetable oil, and vegetable shortening) and salt showed minimal predictive relevance. Differences among protein ingredients may partly relate to their distinct hydration responses, Haleema and Sakhare (2025) reported increased farinograph water absorption with soy protein supplementation, whereas whey and egg protein sources exhibited decreasing trends. Their results illustrate that different protein ingredients can produce distinct hydration responses in wheat flour systems, providing context for the differences in predictive importance observed here. Nevertheless, lower importance does not imply that an ingredient is functionally inactive; it indicates that the model relied less on that variable within the investigated formulation space. Overall, the model relied more strongly on several protein‐related formulation variables than on lipid and salt variables within the tested formulation space. Notably, among the protein sources examined, the tested plant‐derived protein ingredients generally exhibited higher average predictive importance than the tested animal‐derived ingredients. This comparison is restricted to the commercial products examined, because protein origin could not be separated from differences in purity, composition, processing history, particle size, or additives. Furthermore, the averaged ranking may obscure differences among individual mechanical properties and should not be interpreted as evidence that plant‐derived proteins exert universally greater mechanical effects.

#### 3.1.3 Sensitivity of Regression Model Performance to Sequential Ingredient Removal

To evaluate the robustness of formulation‐based prediction and identify the minimal number of predictors necessary to retain model performance, the sensitivity of regression models to sequential ingredient removal was examined for each of the 19 mechanical properties (Supplementary Table 2). Predictive performance was classified into three categories based on the percentage of full‐model *R* ^2^ retained at each removal step: no effect (≥ 90% retention), weakened (70%–90% retention), and damaged (< 70% retention). The ingredients triggering the onset of weakened and damaged performance for each mechanical property are summarized in Table 3.

**TABLE 3:** Sensitivity of regression model performance to sequential ingredient removal across 19 mechanical properties of wheat flour–protein dough.

Property	Ingredient triggering “weakened”	Ingredient triggering “damaged”	Retention at 1 ingredient remaining	Min. ingredients retained (no meaningful change)
Hardness	Pea protein (3 left, 77.2%)	Pea and quinoa protein (2 left, 65.5%)	38.4%	4
Adhesiveness	Whey protein (8 left, 77.7%)	Bread flour (1 left, 10.3%)	10.3%	9
Springiness	Casein protein (9 left, 59.1%)	Casein protein (9 left, 59.1%)	−4.2%	10
Cohesiveness	Casein protein (9 left, 68.3%)	Casein protein (9 left, 68.3%)	−9%	10
Gumminess	Pea protein (3 left, 86.2%)	Bread flour (1 left, 52.5%)	52.5%	4
Chewiness	Pea protein (3 left, 83.7%)	Bread flour (1 left, 49.5%)	49.5%	4
Resilience	Pea protein (3 left, 89.9%)	Bread flour (1 left, 24.9%)	24.9%	4
Storage modulus_Low	Casein protein (9 left, 86.3%)	Pea protein (3 left, 57.9%)	32.6%	10
Storage modulus_Medium	Casein protein (9 left, 87.4%)	Pea protein (3 left, 60.7%)	32.8%	10
Storage modulus_High	Casein protein (9 left, 84.8%)	Pea protein (3 left, 59.4%)	29.9%	10
Loss modulus_Low	Casein protein (9 left, 77.9%)	Water (5 left, 67.9%)	22.7%	10
Loss modulus_Medium	Casein protein (9 left, 78.1%)	Water (5 left, 62.2%)	17.6%	10
Loss modulus_High	Casein protein (9 left, 86.5%)	Egg white protein (7 left, 69%)	7.4%	10
Tan_low	Egg white Protein (7 left, 86.1%)	Pea protein (3 left, 64.6%)	16.9%	8
Tan_Medium	Whey protein (8 left, 88.3%)	Gluten protein (4 left, 61.2%)	13.4%	9
Tan_High	Whey protein (8 left, 89%)	Gluten protein (4 left, 58.4%)	10.6%	9
Complex viscosity_Low	Casein protein (9 left, 86.3%)	Pea protein (3 left, 60.5%)	35.4%	10
Complex viscosity_Medium	Casein protein (9 left, 84.8%)	Pea protein (3 left, 58.6%)	32.1%	10
Complex viscosity_High	Casein protein (9 left, 81.8%)	Gluten protein (4 left, 69.1%)	24%	10

*Note:* Model performance retention was calculated as *R* ^2^_Retention_vs_FullModel (%) = (*R* ^2^ at a given removal step/*R* ^2^ of the full 14‐ingredient model) × 100. Based on this retention percentage, predictive performance was classified as weakened (70%–90% retention) or damaged (< 70% retention).

According to Table 3, several texture properties—including hardness, gumminess, chewiness, and resilience—maintained at least 90% of full‐model predictive performance unaffected predictive performance until only 4 ingredients remained, with pea protein consistently identified as the ingredient whose removal first weakened model performance. After its removal, retention declined to 77.2%, 86.2%, 83.7%, and 89.9%, respectively, indicating that these properties differed in their sensitivity even though they shared the same retained‐variable count. Hardness showed the largest reduction at this step, whereas resilience only marginally crossed the predefined 90% threshold. These differences are descriptive and should not be interpreted as statistically significant without an accompanying uncertainty analysis. In contrast, springiness and cohesiveness were considerably more sensitive to ingredient removal, requiring 10 ingredients to maintain ≥ 90% retention, with casein protein triggering the onset of performance deterioration in both cases. Adhesiveness also required a comparatively broad predictor set, retaining nine ingredients to meet this criterion. Thus, robustness to predictor removal was property‐specific rather than uniform across texture attributes. Notably, springiness and cohesiveness showed negative retention values (springiness: −4.2%; cohesiveness: −9%) when reduced to a single ingredient, indicating that the single‐ingredient model performed worse than a null baseline. Moreover, retaining a high percentage of full‐model *R* ^2^ does not necessarily imply high absolute predictive accuracy, particularly for properties with relatively low full‐model *R* ^2^, such as springiness and cohesiveness (Table 2).

Rheological properties generally required a larger number of ingredients (8–10) to maintain ≥ 90% of full‐model predictive performance compared with most texture properties, with casein protein most frequently identified as the ingredient triggering weakened performance across storage modulus, loss modulus, and complex viscosity at multiple frequency regions. Specifically, all storage modulus, loss modulus, and complex viscosity responses required 10 retained predictors, whereas tan δ required eight at low frequency and nine at medium and high frequencies. Casein removal reduced retention to 84.8%–87.4% for storage modulus, 77.9%–86.5% for loss modulus, and 81.8%–86.3% for complex viscosity. This recurring pattern indicates that casein provided useful predictive information once other variables had already been excluded, despite its relatively low overall importance in Figure 1. Global importance and sequential‐removal sensitivity therefore describe different aspects of predictive relevance: the former summarizes importance across outputs, whereas the latter evaluates the information available within a progressively reduced predictor set.

Previous experimental findings provide context for the involvement of dairy proteins in these responses. Gani et al. (2015) reported that incorporation of whey and casein protein concentrates and their hydrolysates altered wheat dough water absorption, mixing characteristics, and extensibility, with responses depending on supplementation level. These observations support the plausibility that dairy‐protein variables capture formulation‐dependent mechanical variation, but do not directly validate the removal thresholds identified here. Likewise, Roccia et al. (2009) demonstrated that soy protein incorporation affected hydrated gluten through structural interference and reduced water availability, supporting the broader relevance of protein composition and hydration to dough mechanics. Because water addition was adjusted according to formulation workability in the present study, these effects cannot be separated solely through predictor removal.

Frequency‐related differences were also evident after reduction to a single predictor. Loss modulus retention decreased from 22.7% at low frequency to 7.4% at high frequency, whereas storage modulus retained a narrower range of 29.9%–32.8%. Tan(δ) and complex viscosity also showed lower single‐predictor retention at higher frequencies. These results indicate that severe predictor reduction affected the measured responses differently across deformation timescales, although they do not establish a molecular explanation for this pattern.

Taken together, these findings suggest that although rheological properties showed higher absolute predictive accuracy (Section 3.1.1), they were relatively more dependent on retaining a broader set of formulation predictors, whereas several texture properties could be predicted with reasonable robustness using a smaller ingredient subset. Here, ingredient removal refers exclusively to excluding formulation variables from the model, rather than physically removing ingredients from the dough. The identified triggering ingredients and minimum predictor counts are therefore conditional on the removal sequence, model, and tested formulation space, and should not be interpreted as minimum recipe requirements or evidence of causal ingredient effects.

### 3.2 Quantitative Control of Mechanical Properties

#### 3.2.1 Classification Models Predicting Wheat Flour Dough's Mechanical Properties

Classification models utilizing logistic regression, SVC_RBF, random forest, extra trees, and histogram‐based gradient boosting were used to predict the categorical levels (low, medium, and high) of wheat flour–protein dough's mechanical properties, with predictive performance evaluated across all 19 texture and rheological properties using mean cross‐validated prediction accuracy (Table 4).

**TABLE 4:** Predictive performance (mean cross‐validated prediction accuracy) of five classification models for wheat flour–protein dough's texture and rheological properties.

Mechanical properties	Logistic regression	SVC_RBF	Random forest	Extra trees	Hist gradient boosting
Hardness	**0.743**	0.704	0.722	0.701	0.695
Adhesiveness	**0.650**	0.639	0.648	0.646	0.620
Springiness	0.521	0.530	**0.546**	0.537	0.544
Cohesiveness	0.583	**0.627**	0.611	0.616	0.614
Gumminess	**0.699**	0.676	0.669	0.671	0.639
Chewiness	0.630	0.641	0.629	**0.650**	0.592
Resilience	**0.829**	0.764	0.778	0.780	0.766
Storage modulus_Low	**0.738**	0.713	0.667	0.687	0.685
Storage modulus_Medium	**0.725**	0.683	0.667	0.648	0.630
Storage modulus_High	**0.704**	0.646	0.627	0.630	0.609
Loss modulus_Low	**0.731**	0.655	0.637	0.646	0.634
Loss modulus_Medium	**0.685**	0.623	0.581	0.590	0.574
Loss Modulus_High	**0.662**	0.620	0.595	0.606	0.551
Tan (δ)_Low	**0.843**	0.787	0.766	0.787	0.759
Tan (δ)_Medium	**0.884**	0.856	0.822	0.835	0.829
Tan (δ)_High	**0.893**	0.877	0.845	0.868	0.863
Complex viscosity_Low	**0.720**	0.706	0.662	0.674	0.653
Complex viscosity_Medium	**0.722**	0.685	0.653	0.678	0.646
Complex viscosity_High	**0.690**	0.653	0.639	0.630	0.593

*Note:* Bold values indicate the highest mean cross‐validated prediction accuracy within each row. This formatting identifies the numerical maximum only and does not indicate a statistically significant difference between models.

According to Table 4, logistic regression achieved the highest overall predictive performance among the five classification models, consistent with the trend observed for regression models, in which the linear model (Ridge) also performed best (Section 3.1.1). Logistic regression achieved the highest prediction accuracy for 16 of the 19 mechanical properties, including hardness (0.743), gumminess (0.699), resilience (0.829), all three storage modulus frequency regions (0.704–0.738), loss modulus at low and medium frequency (0.685–0.731), and tan(δ) across all frequency regions, reaching as high as 0.893 for tan(δ) at high frequency. The strong performance of logistic regression suggests that comparatively simple decision boundaries could capture useful distinctions among categorical mechanical responses for many properties within the tested formulation space. However, this does not establish that the underlying physical relationships are linear, because classification evaluates separation among tertile‐defined categories rather than the complete continuous response. In contrast, SVC_RBF and extra trees occasionally outperformed logistic regression for a small number of properties, such as cohesiveness (SVC_RBF, 0.627) and chewiness (extra trees, 0.650), while random forest achieved the highest accuracy for springiness (0.546). These exceptions indicate property‐dependent differences in model suitability. Nevertheless, the differences between the highest and second‐highest accuracies for springiness, cohesiveness, and chewiness were only 0.002, 0.011, and 0.009, respectively. Without uncertainty estimates or paired statistical comparisons, these numerical advantages should not be interpreted as statistically significant model superiority.

As with the regression results, classification performance was generally higher for rheological properties—particularly tan(δ), which achieved prediction accuracy above 0.75 across all models and frequency regions—compared with several texture properties such as springiness (0.521–0.546) and cohesiveness (0.583–0.627), which remained comparatively difficult to classify regardless of model choice. Resilience was an important exception, achieving accuracies of 0.764–0.829, demonstrating that texture properties were not uniformly less predictable. Frequency‐dependent trends also differed among rheological responses: logistic regression accuracy declined from low to high frequency for storage modulus (0.738 to 0.704) and loss modulus (0.731 to 0.662), whereas tan(δ) accuracy increased from 0.843 to 0.893. Thus, increasing frequency did not produce a uniform change in predictability across rheological properties. This agreement between the regression and classification results suggests that formulation composition provides useful predictive information for rheological properties, although these variables were less informative for classifying certain texture properties.

Previous experimental studies provide a physical context for these formulation‐dependent responses. Roccia et al. (2009) reported that soy protein incorporation modified hydrated gluten through structural interference and reduced water availability for gluten network formation. Gani et al. (2015) likewise found that whey and casein protein concentrates and their hydrolysates altered wheat dough hydration, mixing characteristics, and extensibility in a supplementation‐dependent manner. These studies support the relevance of protein composition and hydration to dough mechanical behavior, but do not directly explain the relative performance of the classification algorithms. Moreover, classification accuracy reflects the ability to distinguish dataset‐specific low, medium, and high categories; it should not be interpreted as equivalent accuracy in predicting continuous mechanical values or as evidence of externally validated product‐quality thresholds.

#### 3.2.2 Classification‐Based Ingredient Importance Ranking

Permutation importance analysis derived from the classification models revealed a feature relevance hierarchy broadly consistent with the regression‐based findings (Section 3.2.1). This predictive hierarchy is systematically illustrated in Figure 2, which ranks the 14 formulation ingredients based on their mean normalized importance across all modeled categorical outcomes. As demonstrated by the empirical distribution in Figure 2, soy protein was consistently selected as the premier predictive feature, followed by the pea‐and‐quinoa protein mixture and bread flour as the next most weighted predictors, and then by gluten and pea proteins. Together, these five ingredients accounted for the bulk of the overall classification‐based importance.

**FIGURE 2:**
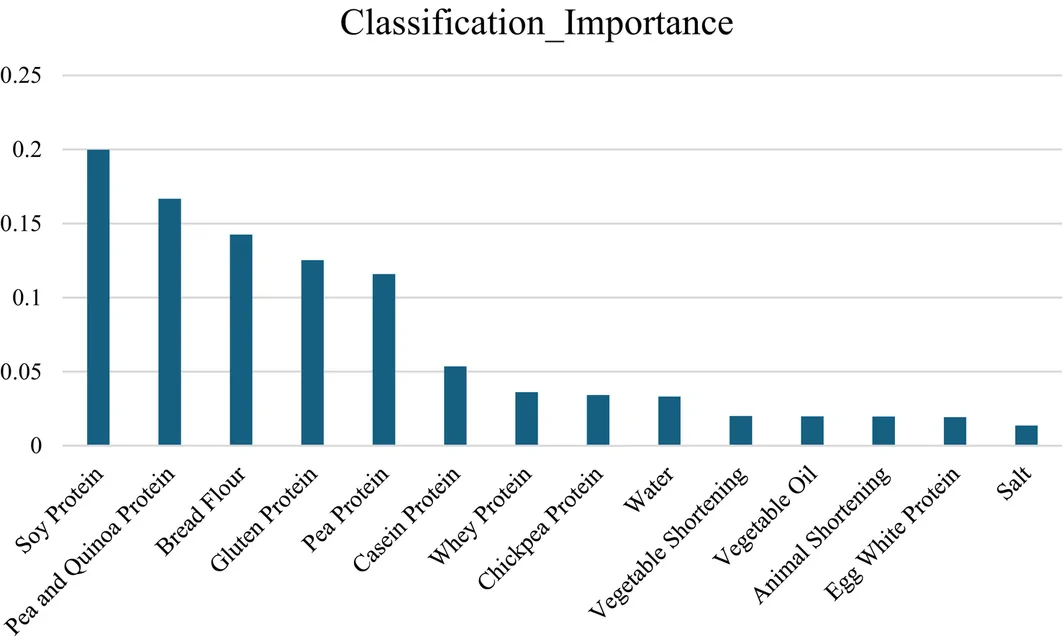
Permutation‐based importance ranking of formulation ingredients using an Extra Trees classifier to predict the categorical levels (low, medium, and high) of 19 dough mechanical properties. Importance was calculated as the decrease in validation balanced accuracy after randomly permuting each predictor. Scores were normalized within each mechanical property to sum to 1.0 and subsequently averaged across all 19 texture and rheological properties. Horizontal bars represent mean normalized importance. Near‐zero values indicate limited average predictive contribution within the tested formulation space, rather than an absence of physical effects.

Beyond these top five ingredients, casein protein ranked sixth, displaying a notably higher predictive weight in classification compared to its regression‐based ranking (Figure 2). In a modeling context, this discrepancy suggests that casein's limited networking‐forming capacity is statistically associated with gluten network disruption in a threshold‐dependent, rather than continuously dose‐dependent manner. Such step‐like predictive relationships are inherently more readily captured by classification algorithms than continuous regression frameworks (Wouters et al. 2016). Conversely, chickpea protein displayed negligible predictive utility in the classification model, despite exhibiting a more substantial statistical association in the regression model. Aside from these two model‐dependent exceptions, the remaining ingredients—whey protein, water, vegetable shortening, vegetable oil, animal shortening, egg white protein, and salt—exhibited only minor predictive contributions across both model tasks.

Overall, the classification‐based ranking closely mirrored the regression‐based ranking in identifying soy protein, the pea‐and‐quinoa protein mixture, bread flour, and pea protein as the dominant contributors to model predictions, while lipid‐related ingredients and salt consistently showed minimal importance across both tasks. This consistency between regression and classification importance rankings suggests that the relative contribution of individual ingredients to dough mechanical properties is robust across different prediction frameworks, whether the outcome is modeled as a continuous variable or as a categorical class. To maintain methodological rigor, we emphasize that the feature importance hierarchies obtained via permutation analysis reflect model‐based predictive associations within the examined formulation space, rather than demonstrating direct biophysical causation. Although these statistical weightings strongly identify which ingredients serve as the most critical predictive markers for dough mechanical behavior, validating the precise molecular interactions and physical causality still requires targeted microstructural and interfacial investigations in future research.

#### 3.2.3 Sensitivity of Classification Model Performance to Sequential Ingredient Removal

To evaluate the robustness of formulation‐based classification and identify the minimal ingredient composition necessary to maintain reliable model performance, the sensitivity of classification models to sequential predictor removal was examined for each of the 19 mechanical properties (Supplementary Table 3). Predictive performance was classified as unaffected (≥ 90% retention), weakened (70%–90% retention), or damaged (< 70% retention) based on the percentage of full‐model balanced accuracy retained at each removal step. The ingredients triggering the onset of weakened and damaged performance for each mechanical property are summarized in Table 5.

**TABLE 5:** Sensitivity of classification model performance to sequential ingredient removal across 19 mechanical properties of wheat flour–protein dough.

Property	Ingredient triggering “weakened”	Ingredient triggering “damaged”	Retention at 1 ingredient remaining	Min. ingredients retained (no meaningful change)
Hardness	Pea protein (4 left, 88.2%)	Not triggered	72.3%	5
Adhesiveness	Pea protein (4 left, 88.2%)	Not triggered	88.6%	3
Springiness	Pea‐quinoa (3 left, 86.2%)	Gluten (1 left, 60.9%)	60.9%	4
Cohesiveness	Pea protein (4 left, 85.7%)	Not triggered	74.2%	5
Gumminess	Pea protein (4 left, 89.7%)	Not triggered	81.1%	5
Chewiness	Gluten (1 left, 87.5%)	Not triggered	87.5%	2
Resilience	Pea protein (4 left, 88.5%)	Not triggered	82.9%	5
Storage modulus_Low	Pea protein (4 left, 88.4%)	Gluten (1 left, 66.1%)	66.1%	5
Storage modulus_Medium	Pea protein (4 left, 87.2%)	Gluten (1 left, 65.2%)	65.2%	5
Storage modulus_High	Casein (6 left, 87.5%)	Gluten (1 left, 61.2%)	61.2%	7
Loss modulus_Low	Casein (6 left, 87.3%)	Gluten (1 left, 60.8%)	60.8%	7
Loss modulus_Medium	Casein (6 left, 87.5%)	Pea‐quinoa (3 left, 69.9%)	57.4%	7
Loss modulus_High	Casein (6 left, 88.8%)	Pea‐quinoa (3 left, 60.1%)	49.6%	7
Tan δ_Low	Pea protein (4 left, 87.7%)	Not triggered	76.7%	5
Tan δ_Medium	Whey protein (7 left, 88.7%)	Gluten (1 left, 68.3%)	68.3%	8
Tan δ_High	Water (5 left, 88.1%)	Gluten (1 left, 67.6%)	67.6%	6
Complex viscosity_Low	Pea‐quinoa (3 left, 83.3%)	Gluten (1 left, 67.2%)	67.2%	4
Complex viscosity_Medium	Pea protein (4 left, 86.2%)	Gluten (1 left, 65.7%)	65.7%	5
Complex viscosity_High	Water (5 left, 87.9%)	Gluten (1 left, 63.4%)	63.4%	6

*Note:* Model‐performance retention was calculated as Balanced Accuracy Retention (%) = (mean cross‐validated balanced accuracy at a given predictor‐removal step/mean cross‐validated balanced accuracy of the full 14‐predictor model) × 100. Performance was classified as unchanged (≥ 90% retention), weakened (70%–90% retention), or damaged (< 70% retention). “Not triggered” indicates that the corresponding threshold was not crossed during the sequential removal procedure. Predictor removal refers to the exclusion of formulation variables from the model rather than physical removal of ingredients from the dough.

According to Table 5, several texture properties—including adhesiveness, gumminess, chewiness, and resilience—maintained comparatively robust predictive performance during sequential predictor removal, although performance eventually declined below the predefined 90% retention threshold. Even when reduced to a single predictor, these properties retained 81.1%–88.6% of full‐model balanced accuracy. Notably, chewiness required as few as two predictors to preserve unchanged performance, indicating that its classification was particularly robust to predictor removal. Adhesiveness and gumminess required three predictors, whereas hardness, cohesiveness, and resilience required five. In contrast, springiness showed greater sensitivity, requiring four predictors to maintain unchanged performance and showing the lowest single‐predictor retention (60.9%) among all texture properties. Pea protein was most frequently identified as the predictor whose removal first weakened performance across the texture properties, whereas the removal of gluten protein caused the largest deterioration for springiness.

Rheological properties generally exhibited greater sensitivity to predictor removal than most texture properties, requiring 4–8 predictors to maintain unchanged predictive performance. Casein protein and pea protein were frequently identified as predictors whose removal first weakened performance across storage modulus, loss modulus, tan(δ), and complex viscosity, whereas gluten protein was most frequently associated with the transition to damaged performance under severe predictor reduction. The pea–quinoa protein blend also triggered the onset of damaged performance for loss modulus at medium and high frequencies. Loss modulus at high frequency was the most sensitive property overall, retaining only 49.6% of full‐model balanced accuracy when reduced to a single predictor. Taken together, these findings parallel those observed for regression models (Section 3.1.3), further supporting the conclusion that rheological properties are generally more dependent on retaining a broader set of formulation predictors, whereas several texture properties can be classified with reasonable robustness using a smaller predictor subset.

### 3.3 Limitations and Generalizability

While the current combinatorial modeling framework successfully elucidates the predictive hierarchy of dietary proteins in dough systems, several inherent limitations and boundaries of generalizability should be explicitly acknowledged:

1. Formulation Domain and Protein Scope: Although 432 formulations represent a comprehensive combinatorial matrix, the experimental domain was restricted to eight conventional plant‐ and animal‐derived protein isolates/concentrates. Furthermore, individual substitution ranges (up to 20.0%–61.1%) and hydration levels were constrained by empirical dough machinability to ensure physical integrity. Consequently, the predictive relationships identified here may not directly extrapolate to novel alternative proteins (e.g., precision fermentation, fungal, or insect proteins) or non‐traditional processing matrices (e.g., gluten‐free systems) without recalibrating the feature space.
2. Intrinsic Multicollinearity and Compositional Constraints: Food dough matrices are inherently compositional systems where relative ingredient fractions sum to unity (the “closure” effect). More importantly, because water addition was adjusted dynamically to offset the disparate water absorption capacities of different proteins, moisture content naturally co‐varied with protein type and concentration. Although regularized linear models (Ridge) and decision‐tree architectures were specifically implemented to mitigate variance inflation caused by correlated predictors, the inherent coupling between hydration demand and protein functionality remains a statistical reality of realistic formulation datasets.
3. Lack of External and Industrial Validation: Model evaluation in this study relied entirely on internal cross‐validation and partitioned test sets generated under strictly standardized, single‐laboratory conditions using a single wheat flour cultivar. The absence of external validation across varied wheat crop years, commercial flour blends, diverse mixing equipment (e.g., industrial spiral or high‐speed continuous mixers), or commercial bakery lines represents a clear boundary. Future work will focus on testing the domain adaptation and transferability of these algorithms in commercial‐scale production environments.
4. Tertile‐Based Discretization Artifacts: For the qualitative classification models, target textural and rheological properties were categorized into discrete classes (low, medium, high) based on empirical tertiles (33.3rd and 66.7th percentiles). While tertile binning establishes balanced class distributions and provides intuitive operational benchmarks for food processors, artificial discretization inherently discards subtle intra‐class continuous variance. Furthermore, observations lying immediately adjacent to class cutoff thresholds risk arbitrary misclassification. Alternative approaches, such as fuzzy logic classification or multi‐objective Pareto optimization, could be integrated in future iterations to alleviate hard boundary artifacts.

## 4 Conclusions

Taken together, the modeling and sequential ingredient‐removal results reveal property‐dependent differences in prediction performance and sensitivity to predictor removal across rheological and texture outputs. Across 432 formulations and 19 mechanical properties, mean cross‐validated regression *R* ^2^ reached 0.865 for tan(δ) at medium frequency, while classification accuracy reached 0.893 for tan(δ) at high frequency. In contrast, springiness and cohesiveness remained comparatively difficult to predict, with maximum *R* ^2^ values of 0.278 and 0.402, respectively. In continuous regression models predicting rheological properties, protein‐related variables collectively demonstrated strong predictive utility, while retaining at least 90% of full‐model *R* ^2^ required eight to 10 formulation predictors, indicating that useful predictive information was distributed across a broader predictor set rather than adequately represented by any isolated ingredient variable. Conversely, for categorical classification of several textural attributes, model performance displayed a comparatively weaker dependence on the complete predictor set. Chewiness retained at least 90% of full‐model classification accuracy with only two predictors, while adhesiveness, gumminess, chewiness, and resilience retained 81.1%–88.6% of full‐model accuracy when reduced to a single predictor. For regression, hardness, gumminess, chewiness, and resilience retained at least 90% of full‐model *R* ^2^ with four predictors. Consistent with this pattern, lipid ingredients (animal shortening, vegetable oil and vegetable shortening) and salt exhibited comparatively low average permutation importance across both quantitative regression and qualitative classification frameworks, supporting the prominent predictive relevance of protein‐related formulation variables within the tested formulation space. Nevertheless, low average importance did not imply that a variable could be omitted without affecting every output.

Within this protein‐focused predictor space, the tested plant‐derived protein ingredients generally exhibited higher average predictive importance than the tested animal‐derived ingredients, with soy protein, bread flour, and the pea–quinoa protein mixture emerging as leading predictors. Rather than demonstrating an inherent biophysical superiority or regulatory role, this divergence may be consistent with gluten‐dilution and competitive hydration effects within the investigated formulations, although these mechanisms were not directly established by the models. The comparison is restricted to the commercial products examined because protein origin could not be separated from differences in ingredient composition and processing history or formulation‐dependent hydration. This pattern was not universal, however; notable model‐dependent divergences emerged, such as casein exhibiting elevated predictive weighting in classification relative to regression, whereas chickpea protein contributed more strongly to continuous regression but showed negligible predictive weight in classification. Casein removal also marked the onset of regression performance deterioration for storage modulus, loss modulus, and complex viscosity across all three frequency regions, with retention declining to 77.9%–87.4%. This indicates that its predictive relevance depended on the target property and the remaining predictor set, despite its relatively low overall regression importance.

Importantly, the findings demonstrate that while empirical machine‐learning models do not establish direct biophysical causation or provide precise processing control, they offer internally validated predictive approximations and valuable screening guidance for multi‐component composite dough formulations within the tested formulation space. Sequential ingredient removal refers to excluding predictors from the models rather than physically removing ingredients from the dough; the retained predictor counts are conditional on the model and removal sequence and therefore do not constitute minimum recipe requirements. Future studies integrating external validation, targeted microstructural characterization, and real‐time mixing kinetics will be critical to translate these predictive associations into validated industrial dough‐processing solutions.

## Author Contributions

**Jingxian An:** writing – original draft. **Owen Jones:** investigation. **Senay Simsek:** supervision. **Kristin Whitney:** investigation. **Bruce Hamaker:** supervision. **Yuan Yao:** funding acquisition, supervision.

## Funding

The authors greatly thank the Whistler Center for Carbohydrate Research for financial support.

## Disclosure

Author responsibility statement: All authors have read and approved the final version of the manuscript. Jingxian An had full access to all of the data in this study and takes complete responsibility for the integrity of the data and the accuracy of the data analysis.

## Ethics Statement

This study does not involve any human or animal testing.

## Consent

The authors have nothing to report.

## Conflicts of Interest

The authors declare no conflicts of interest.

## Supporting information


**Supplementary Table 1.** Ingredient addition ranges expressed as baker's percentages (relative to total flour blend weight or dough weight).
**Supplementary Table 2.** Mean cross‐validated *R* ^2^ (Ridge regression) at each sequential ingredient‐removal step for all 19 mechanical properties of wheat flour–protein dough.
**Supplementary Table 3.** Mean cross‐validated balanced accuracy (logistic regression) at each sequential ingredient‐removal step for all 19 mechanical properties of wheat flour–protein dough.

## Data Availability

The data that support the findings of this study are available from the corresponding author upon reasonable request.
