# Supplementary material for: Quantitative and Qualitative Control of Wheat Flour Dough's Mechanical Properties via Dietary Protein Addition
Source: Food Sci Nutr. 2026 Nov 4;14(11):e72564. doi: 10.1002/fsn3.72564 (PMC13736157; doi:10.1002/fsn3.72564)
Supplement: Supplementary file 1 [file FSN3-14-e72564-s001.docx]

**Supplementary Table 1.** Ingredient addition ranges expressed as baker’s percentages (relative to total flour blend weight or dough weight).

| **Parameter** | **Basis** | **Range** | **Additional statistics** |
| --- | --- | --- | --- |
| Total flour blend weight (bread flour + protein) | g | 12.4–27.5 | - |
| Dough weight (total, incl. water) | g | 28.9–58.3 | - |
|  |  |  |  |
| Protein substitution (overall dataset, incl. 0%) | % of total flour blend | 0.0–70.6 | mean 30.2% |
| Protein substitution (when protein included) | % of total flour blend | 1.2–70.6 | mean 31.1%; P2.5–P97.5: 2.2–63.2% |
| *– Pea (individual max)* | *% of total flour blend* | *up to 52.3* | *n=77* |
| *– Soy (individual max)* | *% of total flour blend* | *up to 61.1* | *n=77* |
| *– Egg white (individual max)* | *% of total flour blend* | *up to 39.7* | *n=77* |
| *– Casein (individual max)* | *% of total flour blend* | *up to 51.7* | *n=77* |
| *– Whey (individual max)* | *% of total flour blend* | *up to 20.0* | *n=77* |
| *– Gluten (individual max)* | *% of total flour blend* | *up to 60.0* | *n=77* |
| *– Chickpea (individual max)* | *% of total flour blend* | *up to 28.6* | *n=77* |
| *– Pea-quinoa blend (individual max)* | *% of total flour blend* | *up to 49.7* | *n=77* |
|  |  |  |  |
| Lipid addition (overall dataset, incl. 0%) | % of dough weight | 0.0–18.7 | mean 8.5% |
| Lipid addition (when lipid included) | % of dough weight | 0.7–18.7 | mean 8.6% |
|  |  |  |  |
| Salt addition (overall dataset, incl. 0%) | % of dough weight | 0.0–3.0 | mean 1.0% |
| Salt addition (when salt included) | % of dough weight | 0.3–3.0 | mean 1.0% |
|  |  |  |  |
| Water-to-total-flour-blend ratio (protein-free formulations) | ratio | 0.55–0.71 | - |
| Water-to-total-flour-blend ratio (protein-containing formulations) | ratio | 0.38–1.59 | mean 0.86 |
| Water-to-total-flour-blend ratio (overall dataset) | ratio | 0.38–1.59 | mean 0.85 |

*Note: Total flour blend weight = bread flour + protein ingredient(s). Dough weight = total flour blend + lipid(s) + salt + water (water density assumed ≈ 1 g/mL). Protein substitution is expressed relative to total flour blend weight; lipid and salt are expressed relative to total dough weight.*

**Supplementary Table 2.** Mean cross-validated R² (Ridge regression) at each sequential ingredient-removal step for all 19 mechanical properties of wheat flour–protein dough.

| **Mechanical Property** | **Ingredient Removal** | **Ingredients_Remaining_n** | **Ridge_CV_R2 mean** |
| --- | --- | --- | --- |
| Hardness | None (full model) | 14 | 0.6399 |
| Hardness | Salt | 13 | 0.6436 |
| Hardness | Vegetable Oil | 12 | 0.6422 |
| Hardness | Vegetable Shortening | 11 | 0.6416 |
| Hardness | Animal Shortening | 10 | 0.6365 |
| Hardness | Casein Protein | 9 | 0.6075 |
| Hardness | Whey Protein | 8 | 0.6085 |
| Hardness | Egg White Protein | 7 | 0.6093 |
| Hardness | Chickpea Protein | 6 | 0.6082 |
| Hardness | Water | 5 | 0.6032 |
| Hardness | Gluten Protein | 4 | 0.576 |
| Hardness | Pea Protein | 3 | 0.4941 |
| Hardness | Pea and Quinoa Protein | 2 | 0.4192 |
| Hardness | Bread Flour | 1 | 0.2455 |
| Adhesiveness | None (full model) | 14 | 0.4132 |
| Adhesiveness | Salt | 13 | 0.4296 |
| Adhesiveness | Vegetable Oil | 12 | 0.4354 |
| Adhesiveness | Vegetable Shortening | 11 | 0.4395 |
| Adhesiveness | Animal Shortening | 10 | 0.4351 |
| Adhesiveness | Casein Protein | 9 | 0.4097 |
| Adhesiveness | Whey Protein | 8 | 0.3211 |
| Adhesiveness | Egg White Protein | 7 | 0.2951 |
| Adhesiveness | Chickpea Protein | 6 | 0.2981 |
| Adhesiveness | Water | 5 | 0.3 |
| Adhesiveness | Gluten Protein | 4 | 0.3016 |
| Adhesiveness | Pea Protein | 3 | 0.304 |
| Adhesiveness | Pea and Quinoa Protein | 2 | 0.3092 |
| Adhesiveness | Bread Flour | 1 | 0.0424 |
| Springiness | None (full model) | 14 | 0.2583 |
| Springiness | Salt | 13 | 0.2556 |
| Springiness | Vegetable Oil | 12 | 0.249 |
| Springiness | Vegetable Shortening | 11 | 0.2456 |
| Springiness | Animal Shortening | 10 | 0.2405 |
| Springiness | Casein Protein | 9 | 0.1527 |
| Springiness | Whey Protein | 8 | 0.165 |
| Springiness | Egg White Protein | 7 | 0.1579 |
| Springiness | Chickpea Protein | 6 | 0.1468 |
| Springiness | Water | 5 | 0.1417 |
| Springiness | Gluten Protein | 4 | 0.0635 |
| Springiness | Pea Protein | 3 | 0.0693 |
| Springiness | Pea and Quinoa Protein | 2 | -0.0114 |
| Springiness | Bread Flour | 1 | -0.011 |
| Cohesiveness | None (full model) | 14 | 0.351 |
| Cohesiveness | Salt | 13 | 0.3585 |
| Cohesiveness | Vegetable Oil | 12 | 0.3608 |
| Cohesiveness | Vegetable Shortening | 11 | 0.3482 |
| Cohesiveness | Animal Shortening | 10 | 0.3182 |
| Cohesiveness | Casein Protein | 9 | 0.2398 |
| Cohesiveness | Whey Protein | 8 | 0.2405 |
| Cohesiveness | Egg White Protein | 7 | 0.2567 |
| Cohesiveness | Chickpea Protein | 6 | 0.2439 |
| Cohesiveness | Water | 5 | 0.2279 |
| Cohesiveness | Gluten Protein | 4 | 0.1144 |
| Cohesiveness | Pea Protein | 3 | 0.1154 |
| Cohesiveness | Pea and Quinoa Protein | 2 | 0.0232 |
| Cohesiveness | Bread Flour | 1 | -0.0315 |
| Gumminess | None (full model) | 14 | 0.5267 |
| Gumminess | Salt | 13 | 0.5342 |
| Gumminess | Vegetable Oil | 12 | 0.5339 |
| Gumminess | Vegetable Shortening | 11 | 0.5371 |
| Gumminess | Animal Shortening | 10 | 0.5351 |
| Gumminess | Casein Protein | 9 | 0.5324 |
| Gumminess | Whey Protein | 8 | 0.53 |
| Gumminess | Egg White Protein | 7 | 0.5343 |
| Gumminess | Chickpea Protein | 6 | 0.5345 |
| Gumminess | Water | 5 | 0.5383 |
| Gumminess | Gluten Protein | 4 | 0.5288 |
| Gumminess | Pea Protein | 3 | 0.4537 |
| Gumminess | Pea and Quinoa Protein | 2 | 0.4412 |
| Gumminess | Bread Flour | 1 | 0.2765 |
| Chewiness | None (full model) | 14 | 0.4091 |
| Chewiness | Salt | 13 | 0.4132 |
| Chewiness | Vegetable Oil | 12 | 0.4127 |
| Chewiness | Vegetable Shortening | 11 | 0.4143 |
| Chewiness | Animal Shortening | 10 | 0.3982 |
| Chewiness | Casein Protein | 9 | 0.404 |
| Chewiness | Whey Protein | 8 | 0.4046 |
| Chewiness | Egg White Protein | 7 | 0.4065 |
| Chewiness | Chickpea Protein | 6 | 0.4079 |
| Chewiness | Water | 5 | 0.4102 |
| Chewiness | Gluten Protein | 4 | 0.4086 |
| Chewiness | Pea Protein | 3 | 0.3424 |
| Chewiness | Pea and Quinoa Protein | 2 | 0.3439 |
| Chewiness | Bread Flour | 1 | 0.2027 |
| Resilience | None (full model) | 14 | 0.6974 |
| Resilience | Salt | 13 | 0.699 |
| Resilience | Vegetable Oil | 12 | 0.6973 |
| Resilience | Vegetable Shortening | 11 | 0.6961 |
| Resilience | Animal Shortening | 10 | 0.6894 |
| Resilience | Casein Protein | 9 | 0.6939 |
| Resilience | Whey Protein | 8 | 0.679 |
| Resilience | Egg White Protein | 7 | 0.6735 |
| Resilience | Chickpea Protein | 6 | 0.6729 |
| Resilience | Water | 5 | 0.6584 |
| Resilience | Gluten Protein | 4 | 0.6453 |
| Resilience | Pea Protein | 3 | 0.6273 |
| Resilience | Pea and Quinoa Protein | 2 | 0.5978 |
| Resilience | Bread Flour | 1 | 0.1736 |
| Storage modulus_Low | None (full model) | 14 | 0.6954 |
| Storage modulus_Low | Salt | 13 | 0.6977 |
| Storage modulus_Low | Vegetable Oil | 12 | 0.6971 |
| Storage modulus_Low | Vegetable Shortening | 11 | 0.6934 |
| Storage modulus_Low | Animal Shortening | 10 | 0.6952 |
| Storage modulus_Low | Casein Protein | 9 | 0.6002 |
| Storage modulus_Low | Whey Protein | 8 | 0.5995 |
| Storage modulus_Low | Egg White Protein | 7 | 0.5969 |
| Storage modulus_Low | Chickpea Protein | 6 | 0.576 |
| Storage modulus_Low | Water | 5 | 0.5484 |
| Storage modulus_Low | Gluten Protein | 4 | 0.5198 |
| Storage modulus_Low | Pea Protein | 3 | 0.4023 |
| Storage modulus_Low | Pea and Quinoa Protein | 2 | 0.2819 |
| Storage modulus_Low | Bread Flour | 1 | 0.2269 |
| Storage modulus_Medium | None (full model) | 14 | 0.6511 |
| Storage modulus_Medium | Salt | 13 | 0.655 |
| Storage modulus_Medium | Vegetable Oil | 12 | 0.6535 |
| Storage modulus_Medium | Vegetable Shortening | 11 | 0.6519 |
| Storage modulus_Medium | Animal Shortening | 10 | 0.6551 |
| Storage modulus_Medium | Casein Protein | 9 | 0.5693 |
| Storage modulus_Medium | Whey Protein | 8 | 0.572 |
| Storage modulus_Medium | Egg White Protein | 7 | 0.5749 |
| Storage modulus_Medium | Chickpea Protein | 6 | 0.5539 |
| Storage modulus_Medium | Water | 5 | 0.5177 |
| Storage modulus_Medium | Gluten Protein | 4 | 0.492 |
| Storage modulus_Medium | Pea Protein | 3 | 0.3949 |
| Storage modulus_Medium | Pea and Quinoa Protein | 2 | 0.2574 |
| Storage modulus_Medium | Bread Flour | 1 | 0.2134 |
| Storage modulus_High | None (full model) | 14 | 0.6122 |
| Storage modulus_High | Salt | 13 | 0.612 |
| Storage modulus_High | Vegetable Oil | 12 | 0.6079 |
| Storage modulus_High | Vegetable Shortening | 11 | 0.6096 |
| Storage modulus_High | Animal Shortening | 10 | 0.6099 |
| Storage modulus_High | Casein Protein | 9 | 0.519 |
| Storage modulus_High | Whey Protein | 8 | 0.5236 |
| Storage modulus_High | Egg White Protein | 7 | 0.5246 |
| Storage modulus_High | Chickpea Protein | 6 | 0.5001 |
| Storage modulus_High | Water | 5 | 0.4469 |
| Storage modulus_High | Gluten Protein | 4 | 0.4349 |
| Storage modulus_High | Pea Protein | 3 | 0.3637 |
| Storage modulus_High | Pea and Quinoa Protein | 2 | 0.1957 |
| Storage modulus_High | Bread Flour | 1 | 0.1828 |
| Loss modulus_Low | None (full model) | 14 | 0.6536 |
| Loss modulus_Low | Salt | 13 | 0.656 |
| Loss modulus_Low | Vegetable Oil | 12 | 0.6457 |
| Loss modulus_Low | Vegetable Shortening | 11 | 0.6447 |
| Loss modulus_Low | Animal Shortening | 10 | 0.6463 |
| Loss modulus_Low | Casein Protein | 9 | 0.5094 |
| Loss modulus_Low | Whey Protein | 8 | 0.5145 |
| Loss modulus_Low | Egg White Protein | 7 | 0.5165 |
| Loss modulus_Low | Chickpea Protein | 6 | 0.489 |
| Loss modulus_Low | Water | 5 | 0.4435 |
| Loss modulus_Low | Gluten Protein | 4 | 0.4249 |
| Loss modulus_Low | Pea Protein | 3 | 0.3381 |
| Loss modulus_Low | Pea and Quinoa Protein | 2 | 0.163 |
| Loss modulus_Low | Bread Flour | 1 | 0.1484 |
| Loss modulus_Medium | None (full model) | 14 | 0.6012 |
| Loss modulus_Medium | Salt | 13 | 0.6038 |
| Loss modulus_Medium | Vegetable Oil | 12 | 0.5942 |
| Loss modulus_Medium | Vegetable Shortening | 11 | 0.5948 |
| Loss modulus_Medium | Animal Shortening | 10 | 0.5951 |
| Loss modulus_Medium | Casein Protein | 9 | 0.4696 |
| Loss modulus_Medium | Whey Protein | 8 | 0.4691 |
| Loss modulus_Medium | Egg White Protein | 7 | 0.4654 |
| Loss modulus_Medium | Chickpea Protein | 6 | 0.4439 |
| Loss modulus_Medium | Water | 5 | 0.3737 |
| Loss modulus_Medium | Gluten Protein | 4 | 0.365 |
| Loss modulus_Medium | Pea Protein | 3 | 0.2826 |
| Loss modulus_Medium | Pea and Quinoa Protein | 2 | 0.1051 |
| Loss modulus_Medium | Bread Flour | 1 | 0.1058 |
| Loss modulus_High | None (full model) | 14 | 0.4916 |
| Loss modulus_High | Salt | 13 | 0.4946 |
| Loss modulus_High | Vegetable Oil | 12 | 0.4879 |
| Loss modulus_High | Vegetable Shortening | 11 | 0.4907 |
| Loss modulus_High | Animal Shortening | 10 | 0.4961 |
| Loss modulus_High | Casein Protein | 9 | 0.4253 |
| Loss modulus_High | Whey Protein | 8 | 0.4226 |
| Loss modulus_High | Egg White Protein | 7 | 0.3394 |
| Loss modulus_High | Chickpea Protein | 6 | 0.3154 |
| Loss modulus_High | Water | 5 | 0.2332 |
| Loss modulus_High | Gluten Protein | 4 | 0.2311 |
| Loss modulus_High | Pea Protein | 3 | 0.1866 |
| Loss modulus_High | Pea and Quinoa Protein | 2 | 0.0398 |
| Loss modulus_High | Bread Flour | 1 | 0.0364 |
| Tan δ_Low | None (full model) | 14 | 0.7404 |
| Tan δ_Low | Salt | 13 | 0.7319 |
| Tan δ_Low | Vegetable Oil | 12 | 0.7324 |
| Tan δ_Low | Vegetable Shortening | 11 | 0.7254 |
| Tan δ_Low | Animal Shortening | 10 | 0.726 |
| Tan δ_Low | Casein Protein | 9 | 0.6988 |
| Tan δ_Low | Whey Protein | 8 | 0.6714 |
| Tan δ_Low | Egg White Protein | 7 | 0.6373 |
| Tan δ_Low | Chickpea Protein | 6 | 0.6251 |
| Tan δ_Low | Water | 5 | 0.6034 |
| Tan δ_Low | Gluten Protein | 4 | 0.5454 |
| Tan δ_Low | Pea Protein | 3 | 0.4782 |
| Tan δ_Low | Pea and Quinoa Protein | 2 | 0.4716 |
| Tan δ_Low | Bread Flour | 1 | 0.1251 |
| Tan δ_Medium | None (full model) | 14 | 0.8137 |
| Tan δ_Medium | Salt | 13 | 0.8094 |
| Tan δ_Medium | Vegetable Oil | 12 | 0.8095 |
| Tan δ_Medium | Vegetable Shortening | 11 | 0.802 |
| Tan δ_Medium | Animal Shortening | 10 | 0.8018 |
| Tan δ_Medium | Casein Protein | 9 | 0.7822 |
| Tan δ_Medium | Whey Protein | 8 | 0.7186 |
| Tan δ_Medium | Egg White Protein | 7 | 0.6182 |
| Tan δ_Medium | Chickpea Protein | 6 | 0.6121 |
| Tan δ_Medium | Water | 5 | 0.5835 |
| Tan δ_Medium | Gluten Protein | 4 | 0.4977 |
| Tan δ_Medium | Pea Protein | 3 | 0.4506 |
| Tan δ_Medium | Pea and Quinoa Protein | 2 | 0.4451 |
| Tan δ_Medium | Bread Flour | 1 | 0.1087 |
| Tan δ_High | None (full model) | 14 | 0.8588 |
| Tan δ_High | Salt | 13 | 0.8563 |
| Tan δ_High | Vegetable Oil | 12 | 0.8566 |
| Tan δ_High | Vegetable Shortening | 11 | 0.854 |
| Tan δ_High | Animal Shortening | 10 | 0.8543 |
| Tan δ_High | Casein Protein | 9 | 0.8397 |
| Tan δ_High | Whey Protein | 8 | 0.7645 |
| Tan δ_High | Egg White Protein | 7 | 0.6725 |
| Tan δ_High | Chickpea Protein | 6 | 0.6761 |
| Tan δ_High | Water | 5 | 0.6307 |
| Tan δ_High | Gluten Protein | 4 | 0.5019 |
| Tan δ_High | Pea Protein | 3 | 0.4562 |
| Tan δ_High | Pea and Quinoa Protein | 2 | 0.4436 |
| Tan δ_High | Bread Flour | 1 | 0.0911 |
| Complex viscosity_Low | None (full model) | 14 | 0.7008 |
| Complex viscosity_Low | Salt | 13 | 0.7023 |
| Complex viscosity_Low | Vegetable Oil | 12 | 0.7001 |
| Complex viscosity_Low | Vegetable Shortening | 11 | 0.6976 |
| Complex viscosity_Low | Animal Shortening | 10 | 0.7 |
| Complex viscosity_Low | Casein Protein | 9 | 0.605 |
| Complex viscosity_Low | Whey Protein | 8 | 0.6041 |
| Complex viscosity_Low | Egg White Protein | 7 | 0.6033 |
| Complex viscosity_Low | Chickpea Protein | 6 | 0.5809 |
| Complex viscosity_Low | Water | 5 | 0.5503 |
| Complex viscosity_Low | Gluten Protein | 4 | 0.5183 |
| Complex viscosity_Low | Pea Protein | 3 | 0.4238 |
| Complex viscosity_Low | Pea and Quinoa Protein | 2 | 0.3052 |
| Complex viscosity_Low | Bread Flour | 1 | 0.248 |
| Complex viscosity_Medium | None (full model) | 14 | 0.6658 |
| Complex viscosity_Medium | Salt | 13 | 0.6668 |
| Complex viscosity_Medium | Vegetable Oil | 12 | 0.6651 |
| Complex viscosity_Medium | Vegetable Shortening | 11 | 0.6648 |
| Complex viscosity_Medium | Animal Shortening | 10 | 0.6654 |
| Complex viscosity_Medium | Casein Protein | 9 | 0.5649 |
| Complex viscosity_Medium | Whey Protein | 8 | 0.5656 |
| Complex viscosity_Medium | Egg White Protein | 7 | 0.567 |
| Complex viscosity_Medium | Chickpea Protein | 6 | 0.5447 |
| Complex viscosity_Medium | Water | 5 | 0.5123 |
| Complex viscosity_Medium | Gluten Protein | 4 | 0.4888 |
| Complex viscosity_Medium | Pea Protein | 3 | 0.3903 |
| Complex viscosity_Medium | Pea and Quinoa Protein | 2 | 0.2494 |
| Complex viscosity_Medium | Bread Flour | 1 | 0.2136 |
| Complex viscosity_High | None (full model) | 14 | 0.6124 |
| Complex viscosity_High | Salt | 13 | 0.6123 |
| Complex viscosity_High | Vegetable Oil | 12 | 0.6103 |
| Complex viscosity_High | Vegetable Shortening | 11 | 0.6099 |
| Complex viscosity_High | Animal Shortening | 10 | 0.6105 |
| Complex viscosity_High | Casein Protein | 9 | 0.5009 |
| Complex viscosity_High | Whey Protein | 8 | 0.5104 |
| Complex viscosity_High | Egg White Protein | 7 | 0.5113 |
| Complex viscosity_High | Chickpea Protein | 6 | 0.4869 |
| Complex viscosity_High | Water | 5 | 0.435 |
| Complex viscosity_High | Gluten Protein | 4 | 0.4231 |
| Complex viscosity_High | Pea Protein | 3 | 0.328 |
| Complex viscosity_High | Pea and Quinoa Protein | 2 | 0.1608 |
| Complex viscosity_High | Bread Flour | 1 | 0.1472 |

**Note:** Ingredients were removed one at a time in order of increasing importance, as determined by permutation importance recalculated at each step (see Section 2.2.2). "Ingredients_Remaining_n" indicates the number of ingredients retained in the model at each step, and "Ridge_CV_R2 mean" reports the mean R² across repeated 5-fold cross-validation folds for the corresponding step. Full model (Step 0) corresponds to all 14 variable ingredients.

**Supplementary Table 3.** Mean cross-validated balanced accuracy (logistic regression) at each sequential ingredient-removal step for all 19 mechanical properties of wheat flour–protein dough.

| Mechanical Property | Ingredient Removal | Ingredients_Remaining_n | LogReg_CV_Accuracy_mean |
| --- | --- | --- | --- |
| Hardness | None (full model) | 14 | 0.7432 |
| Hardness | Salt | 13 | 0.7339 |
| Hardness | Egg White Protein | 12 | 0.7269 |
| Hardness | Animal Shortening | 11 | 0.7385 |
| Hardness | Vegetable Oil | 10 | 0.7269 |
| Hardness | Vegetable Shortening | 9 | 0.7269 |
| Hardness | Chickpea Protein | 8 | 0.7316 |
| Hardness | Whey Protein | 7 | 0.7176 |
| Hardness | Casein Protein | 6 | 0.7246 |
| Hardness | Water | 5 | 0.7270 |
| Hardness | Pea Protein | 4 | 0.6553 |
| Hardness | Pea and Quinoa Protein | 3 | 0.6438 |
| Hardness | Soy Protein | 2 | 0.6484 |
| Hardness | Gluten Protein | 1 | 0.5372 |
| Adhesiveness | None (full model) | 14 | 0.6504 |
| Adhesiveness | Salt | 13 | 0.6596 |
| Adhesiveness | Egg White Protein | 12 | 0.6365 |
| Adhesiveness | Animal Shortening | 11 | 0.6319 |
| Adhesiveness | Vegetable Oil | 10 | 0.6341 |
| Adhesiveness | Vegetable Shortening | 9 | 0.6225 |
| Adhesiveness | Chickpea Protein | 8 | 0.6365 |
| Adhesiveness | Whey Protein | 7 | 0.6088 |
| Adhesiveness | Casein Protein | 6 | 0.5972 |
| Adhesiveness | Water | 5 | 0.5925 |
| Adhesiveness | Pea Protein | 4 | 0.5809 |
| Adhesiveness | Pea and Quinoa Protein | 3 | 0.5902 |
| Adhesiveness | Soy Protein | 2 | 0.5763 |
| Adhesiveness | Gluten Protein | 1 | 0.5763 |
| Springiness | None (full model) | 14 | 0.5207 |
| Springiness | Salt | 13 | 0.5300 |
| Springiness | Egg White Protein | 12 | 0.5183 |
| Springiness | Animal Shortening | 11 | 0.5044 |
| Springiness | Vegetable Oil | 10 | 0.5090 |
| Springiness | Vegetable Shortening | 9 | 0.5067 |
| Springiness | Chickpea Protein | 8 | 0.5068 |
| Springiness | Whey Protein | 7 | 0.4928 |
| Springiness | Casein Protein | 6 | 0.4883 |
| Springiness | Water | 5 | 0.4859 |
| Springiness | Pea Protein | 4 | 0.4790 |
| Springiness | Pea and Quinoa Protein | 3 | 0.4490 |
| Springiness | Soy Protein | 2 | 0.4166 |
| Springiness | Gluten Protein | 1 | 0.3173 |
| Cohesiveness | None (full model) | 14 | 0.5833 |
| Cohesiveness | Salt | 13 | 0.5903 |
| Cohesiveness | Egg White Protein | 12 | 0.5880 |
| Cohesiveness | Animal Shortening | 11 | 0.5856 |
| Cohesiveness | Vegetable Oil | 10 | 0.5971 |
| Cohesiveness | Vegetable Shortening | 9 | 0.5948 |
| Cohesiveness | Chickpea Protein | 8 | 0.5924 |
| Cohesiveness | Whey Protein | 7 | 0.5855 |
| Cohesiveness | Casein Protein | 6 | 0.6039 |
| Cohesiveness | Water | 5 | 0.5669 |
| Cohesiveness | Pea Protein | 4 | 0.4999 |
| Cohesiveness | Pea and Quinoa Protein | 3 | 0.4999 |
| Cohesiveness | Soy Protein | 2 | 0.4930 |
| Cohesiveness | Gluten Protein | 1 | 0.4328 |
| Gumminess | None (full model) | 14 | 0.6990 |
| Gumminess | Salt | 13 | 0.7037 |
| Gumminess | Egg White Protein | 12 | 0.7175 |
| Gumminess | Animal Shortening | 11 | 0.7082 |
| Gumminess | Vegetable Oil | 10 | 0.7082 |
| Gumminess | Vegetable Shortening | 9 | 0.7105 |
| Gumminess | Chickpea Protein | 8 | 0.6967 |
| Gumminess | Whey Protein | 7 | 0.6827 |
| Gumminess | Casein Protein | 6 | 0.6782 |
| Gumminess | Water | 5 | 0.6804 |
| Gumminess | Pea Protein | 4 | 0.6272 |
| Gumminess | Pea and Quinoa Protein | 3 | 0.6295 |
| Gumminess | Soy Protein | 2 | 0.6110 |
| Gumminess | Gluten Protein | 1 | 0.5671 |
| Chewiness | None (full model) | 14 | 0.6296 |
| Chewiness | Salt | 13 | 0.6434 |
| Chewiness | Egg White Protein | 12 | 0.6410 |
| Chewiness | Animal Shortening | 11 | 0.6317 |
| Chewiness | Vegetable Oil | 10 | 0.6271 |
| Chewiness | Vegetable Shortening | 9 | 0.6272 |
| Chewiness | Chickpea Protein | 8 | 0.6364 |
| Chewiness | Whey Protein | 7 | 0.6296 |
| Chewiness | Casein Protein | 6 | 0.6225 |
| Chewiness | Water | 5 | 0.6133 |
| Chewiness | Pea Protein | 4 | 0.5855 |
| Chewiness | Pea and Quinoa Protein | 3 | 0.6018 |
| Chewiness | Soy Protein | 2 | 0.5879 |
| Chewiness | Gluten Protein | 1 | 0.5509 |
| Resilience | None (full model) | 14 | 0.8288 |
| Resilience | Salt | 13 | 0.8218 |
| Resilience | Egg White Protein | 12 | 0.8195 |
| Resilience | Animal Shortening | 11 | 0.8010 |
| Resilience | Vegetable Oil | 10 | 0.8009 |
| Resilience | Vegetable Shortening | 9 | 0.7963 |
| Resilience | Chickpea Protein | 8 | 0.8010 |
| Resilience | Whey Protein | 7 | 0.7847 |
| Resilience | Casein Protein | 6 | 0.7847 |
| Resilience | Water | 5 | 0.7592 |
| Resilience | Pea Protein | 4 | 0.7337 |
| Resilience | Pea and Quinoa Protein | 3 | 0.7289 |
| Resilience | Soy Protein | 2 | 0.6897 |
| Resilience | Gluten Protein | 1 | 0.6872 |
| Storage modulus_Low | None (full model) | 14 | 0.7384 |
| Storage modulus_Low | Salt | 13 | 0.7499 |
| Storage modulus_Low | Egg White Protein | 12 | 0.7407 |
| Storage modulus_Low | Animal Shortening | 11 | 0.7500 |
| Storage modulus_Low | Vegetable Oil | 10 | 0.7500 |
| Storage modulus_Low | Vegetable Shortening | 9 | 0.7545 |
| Storage modulus_Low | Chickpea Protein | 8 | 0.7245 |
| Storage modulus_Low | Whey Protein | 7 | 0.7197 |
| Storage modulus_Low | Casein Protein | 6 | 0.6919 |
| Storage modulus_Low | Water | 5 | 0.7060 |
| Storage modulus_Low | Pea Protein | 4 | 0.6527 |
| Storage modulus_Low | Pea and Quinoa Protein | 3 | 0.6041 |
| Storage modulus_Low | Soy Protein | 2 | 0.5948 |
| Storage modulus_Low | Gluten Protein | 1 | 0.4882 |
| Storage modulus_Medium | None (full model) | 14 | 0.7245 |
| Storage modulus_Medium | Salt | 13 | 0.7292 |
| Storage modulus_Medium | Egg White Protein | 12 | 0.7130 |
| Storage modulus_Medium | Animal Shortening | 11 | 0.7291 |
| Storage modulus_Medium | Vegetable Oil | 10 | 0.7268 |
| Storage modulus_Medium | Vegetable Shortening | 9 | 0.7292 |
| Storage modulus_Medium | Chickpea Protein | 8 | 0.7037 |
| Storage modulus_Medium | Whey Protein | 7 | 0.6921 |
| Storage modulus_Medium | Casein Protein | 6 | 0.6621 |
| Storage modulus_Medium | Water | 5 | 0.6668 |
| Storage modulus_Medium | Pea Protein | 4 | 0.6321 |
| Storage modulus_Medium | Pea and Quinoa Protein | 3 | 0.5673 |
| Storage modulus_Medium | Soy Protein | 2 | 0.5510 |
| Storage modulus_Medium | Gluten Protein | 1 | 0.4725 |
| Storage modulus_High | None (full model) | 14 | 0.7038 |
| Storage modulus_High | Salt | 13 | 0.7015 |
| Storage modulus_High | Egg White Protein | 12 | 0.6922 |
| Storage modulus_High | Animal Shortening | 11 | 0.6852 |
| Storage modulus_High | Vegetable Oil | 10 | 0.6968 |
| Storage modulus_High | Vegetable Shortening | 9 | 0.6945 |
| Storage modulus_High | Chickpea Protein | 8 | 0.6898 |
| Storage modulus_High | Whey Protein | 7 | 0.6828 |
| Storage modulus_High | Casein Protein | 6 | 0.6157 |
| Storage modulus_High | Water | 5 | 0.5971 |
| Storage modulus_High | Pea Protein | 4 | 0.5950 |
| Storage modulus_High | Pea and Quinoa Protein | 3 | 0.4978 |
| Storage modulus_High | Soy Protein | 2 | 0.5092 |
| Storage modulus_High | Gluten Protein | 1 | 0.4304 |
| Loss modulus_Low | None (full model) | 14 | 0.7313 |
| Loss modulus_Low | Salt | 13 | 0.7336 |
| Loss modulus_Low | Egg White Protein | 12 | 0.7059 |
| Loss modulus_Low | Animal Shortening | 11 | 0.7082 |
| Loss modulus_Low | Vegetable Oil | 10 | 0.7081 |
| Loss modulus_Low | Vegetable Shortening | 9 | 0.7035 |
| Loss modulus_Low | Chickpea Protein | 8 | 0.6827 |
| Loss modulus_Low | Whey Protein | 7 | 0.6827 |
| Loss modulus_Low | Casein Protein | 6 | 0.6387 |
| Loss modulus_Low | Water | 5 | 0.6295 |
| Loss modulus_Low | Pea Protein | 4 | 0.6088 |
| Loss modulus_Low | Pea and Quinoa Protein | 3 | 0.5303 |
| Loss modulus_Low | Soy Protein | 2 | 0.5232 |
| Loss modulus_Low | Gluten Protein | 1 | 0.4445 |
| Loss modulus_Medium | None (full model) | 14 | 0.6853 |
| Loss modulus_Medium | Salt | 13 | 0.6644 |
| Loss modulus_Medium | Egg White Protein | 12 | 0.6713 |
| Loss modulus_Medium | Animal Shortening | 11 | 0.6782 |
| Loss modulus_Medium | Vegetable Oil | 10 | 0.6806 |
| Loss modulus_Medium | Vegetable Shortening | 9 | 0.6759 |
| Loss modulus_Medium | Chickpea Protein | 8 | 0.6505 |
| Loss modulus_Medium | Whey Protein | 7 | 0.6621 |
| Loss modulus_Medium | Casein Protein | 6 | 0.5996 |
| Loss modulus_Medium | Water | 5 | 0.5834 |
| Loss modulus_Medium | Pea Protein | 4 | 0.5603 |
| Loss modulus_Medium | Pea and Quinoa Protein | 3 | 0.4791 |
| Loss modulus_Medium | Soy Protein | 2 | 0.4952 |
| Loss modulus_Medium | Gluten Protein | 1 | 0.3936 |
| Loss modulus_High | None (full model) | 14 | 0.6621 |
| Loss modulus_High | Salt | 13 | 0.6667 |
| Loss modulus_High | Egg White Protein | 12 | 0.6713 |
| Loss modulus_High | Animal Shortening | 11 | 0.6945 |
| Loss modulus_High | Vegetable Oil | 10 | 0.6945 |
| Loss modulus_High | Vegetable Shortening | 9 | 0.6736 |
| Loss modulus_High | Chickpea Protein | 8 | 0.6226 |
| Loss modulus_High | Whey Protein | 7 | 0.6273 |
| Loss modulus_High | Casein Protein | 6 | 0.5878 |
| Loss modulus_High | Water | 5 | 0.5671 |
| Loss modulus_High | Pea Protein | 4 | 0.4745 |
| Loss modulus_High | Pea and Quinoa Protein | 3 | 0.3980 |
| Loss modulus_High | Soy Protein | 2 | 0.3911 |
| Loss modulus_High | Gluten Protein | 1 | 0.3285 |
| Tan δ_Low | None (full model) | 14 | 0.8425 |
| Tan δ_Low | Salt | 13 | 0.8379 |
| Tan δ_Low | Egg White Protein | 12 | 0.8079 |
| Tan δ_Low | Animal Shortening | 11 | 0.8102 |
| Tan δ_Low | Vegetable Oil | 10 | 0.8148 |
| Tan δ_Low | Vegetable Shortening | 9 | 0.8149 |
| Tan δ_Low | Chickpea Protein | 8 | 0.8103 |
| Tan δ_Low | Whey Protein | 7 | 0.7687 |
| Tan δ_Low | Casein Protein | 6 | 0.7687 |
| Tan δ_Low | Water | 5 | 0.7687 |
| Tan δ_Low | Pea Protein | 4 | 0.7386 |
| Tan δ_Low | Pea and Quinoa Protein | 3 | 0.7316 |
| Tan δ_Low | Soy Protein | 2 | 0.6945 |
| Tan δ_Low | Gluten Protein | 1 | 0.6460 |
| Tan δ_Medium | None (full model) | 14 | 0.8842 |
| Tan δ_Medium | Salt | 13 | 0.8773 |
| Tan δ_Medium | Egg White Protein | 12 | 0.8356 |
| Tan δ_Medium | Animal Shortening | 11 | 0.8356 |
| Tan δ_Medium | Vegetable Oil | 10 | 0.8379 |
| Tan δ_Medium | Vegetable Shortening | 9 | 0.8447 |
| Tan δ_Medium | Chickpea Protein | 8 | 0.8309 |
| Tan δ_Medium | Whey Protein | 7 | 0.7845 |
| Tan δ_Medium | Casein Protein | 6 | 0.7916 |
| Tan δ_Medium | Water | 5 | 0.7522 |
| Tan δ_Medium | Pea Protein | 4 | 0.7314 |
| Tan δ_Medium | Pea and Quinoa Protein | 3 | 0.7199 |
| Tan δ_Medium | Soy Protein | 2 | 0.7176 |
| Tan δ_Medium | Gluten Protein | 1 | 0.6043 |
| Tan δ_High | None (full model) | 14 | 0.8933 |
| Tan δ_High | Salt | 13 | 0.8933 |
| Tan δ_High | Egg White Protein | 12 | 0.8702 |
| Tan δ_High | Animal Shortening | 11 | 0.8679 |
| Tan δ_High | Vegetable Oil | 10 | 0.8748 |
| Tan δ_High | Vegetable Shortening | 9 | 0.8771 |
| Tan δ_High | Chickpea Protein | 8 | 0.8656 |
| Tan δ_High | Whey Protein | 7 | 0.8216 |
| Tan δ_High | Casein Protein | 6 | 0.8124 |
| Tan δ_High | Water | 5 | 0.7868 |
| Tan δ_High | Pea Protein | 4 | 0.7729 |
| Tan δ_High | Pea and Quinoa Protein | 3 | 0.7706 |
| Tan δ_High | Soy Protein | 2 | 0.7637 |
| Tan δ_High | Gluten Protein | 1 | 0.6042 |
| Complex viscosity_Low | None (full model) | 14 | 0.7199 |
| Complex viscosity_Low | Salt | 13 | 0.7361 |
| Complex viscosity_Low | Egg White Protein | 12 | 0.7361 |
| Complex viscosity_Low | Animal Shortening | 11 | 0.7338 |
| Complex viscosity_Low | Vegetable Oil | 10 | 0.7454 |
| Complex viscosity_Low | Vegetable Shortening | 9 | 0.7361 |
| Complex viscosity_Low | Chickpea Protein | 8 | 0.7199 |
| Complex viscosity_Low | Whey Protein | 7 | 0.7153 |
| Complex viscosity_Low | Casein Protein | 6 | 0.6876 |
| Complex viscosity_Low | Water | 5 | 0.6829 |
| Complex viscosity_Low | Pea Protein | 4 | 0.6620 |
| Complex viscosity_Low | Pea and Quinoa Protein | 3 | 0.5996 |
| Complex viscosity_Low | Soy Protein | 2 | 0.6018 |
| Complex viscosity_Low | Gluten Protein | 1 | 0.4840 |
| Complex viscosity_Medium | None (full model) | 14 | 0.7221 |
| Complex viscosity_Medium | Salt | 13 | 0.7291 |
| Complex viscosity_Medium | Egg White Protein | 12 | 0.7014 |
| Complex viscosity_Medium | Animal Shortening | 11 | 0.7152 |
| Complex viscosity_Medium | Vegetable Oil | 10 | 0.7082 |
| Complex viscosity_Medium | Vegetable Shortening | 9 | 0.7082 |
| Complex viscosity_Medium | Chickpea Protein | 8 | 0.6989 |
| Complex viscosity_Medium | Whey Protein | 7 | 0.7013 |
| Complex viscosity_Medium | Casein Protein | 6 | 0.6642 |
| Complex viscosity_Medium | Water | 5 | 0.6596 |
| Complex viscosity_Medium | Pea Protein | 4 | 0.6226 |
| Complex viscosity_Medium | Pea and Quinoa Protein | 3 | 0.5510 |
| Complex viscosity_Medium | Soy Protein | 2 | 0.5695 |
| Complex viscosity_Medium | Gluten Protein | 1 | 0.4745 |
| Complex viscosity_High | None (full model) | 14 | 0.6898 |
| Complex viscosity_High | Salt | 13 | 0.6944 |
| Complex viscosity_High | Egg White Protein | 12 | 0.6967 |
| Complex viscosity_High | Animal Shortening | 11 | 0.6851 |
| Complex viscosity_High | Vegetable Oil | 10 | 0.7013 |
| Complex viscosity_High | Vegetable Shortening | 9 | 0.7013 |
| Complex viscosity_High | Chickpea Protein | 8 | 0.6690 |
| Complex viscosity_High | Whey Protein | 7 | 0.6690 |
| Complex viscosity_High | Casein Protein | 6 | 0.6366 |
| Complex viscosity_High | Water | 5 | 0.6065 |
| Complex viscosity_High | Pea Protein | 4 | 0.5879 |
| Complex viscosity_High | Pea and Quinoa Protein | 3 | 0.5070 |
| Complex viscosity_High | Soy Protein | 2 | 0.5022 |
| Complex viscosity_High | Gluten Protein | 1 | 0.4376 |

**Note:** Ingredients were removed one at a time in order of increasing classification-based importance, as determined by permutation importance recalculated at each step (see Section 2.2.2). "Ingredients_Remaining_n" indicates the number of ingredients retained in the model at each step, and "LogReg_CV_Accuracy_mean" reports the mean balanced accuracy across repeated 5-fold cross-validation folds for the corresponding step. Full model (Step 0) corresponds to all 14 variable ingredients.
